# The Non-Panel Movement

**Published:** 1913-11-08

**Authors:** 


### THE NON-PANEL MOVEMENT.

### The Moral of Wisbech and Chesterfield.

During the past week there have been meetings of practitioners in Lambeth, St. Pancras, and Islington, and other districts, at which resolutions to form non-panel associations have been passed. These newly formed societies will doubtless be entitled to representation at the conference now being arranged by the National Medical Union, which, as already announced, will shortly be held in London. Next week, the Edinburgh Guild of Non-Panel Practitioners is to hold a meeting which will be attended by representatives of other non-panel associations, and at which questions of future procedure will be discussed.

### The B.M.A. and Non-Panel Members.

Some interest attaches to the announcement in the *British Medical Journal* that the Council of the Association has appointed a special committee to consider the interests of non-panel members. This step is no doubt the outcome of threatened and actual resignations from the Association, and represents a natural attempt to retain or regain the seceding members. It is doubtful, however, if the new body will be capable of effective action in the intended direction, as it is appointed with the following reference: "To consider in what manner the British Medical Association can best promote the interests of members who have not entered into agreements with Insurance Commitees."

It appears from a consideration of the terms of reference that any recommendation of the new committee must be made to the Council and acted on, if at all, by that body, which is already under a decided suspicion of being pro-panel in its leanings. It must be recollected, too, that the Council's recommendations on all matters of moment must be referred to the divisions, and must subsequently come before a representative meeting for confirmation before any action can be taken. This slow and cumbrous procedure has been fatal to effective action in the past, and is likely to prove equally obstructive in the future until the constitution of the Association is radically altered. The non-panel societies now existing or in process of formation may thus be expected to find the progress of the movement hindered rather than assisted by the British Medical Association influence; and they will probably proceed with their programmes without waiting to see what results, if any, are obtained through the action of the new committee. For there is no denying that panel and non-panel interests are incompatible on so many points that those are doubtless right who argue that the Association cannot continue to represent both classes of members, and that having declared for the panel—in practice, if not in theory—it should devote itself to the panel members and leave the non-panel men to form and federate associations of their own.

The activity of the non-panel movement is being stimulated by the deepening conviction that all is not well with the panel system, despite the stereotyped assurance so often given that the Insurance Act is "working smoothly."

Now and then, as last week for instance, the forces beneath the surface rise to give very practical proof of the real state of things. The recent disturbances at Wisbech, whatever their immediate causes, can only be regarded as a very disquieting by-product, so to speak, of the faulty administration of the Act. Here we have a district where all might have gone smoothly had not the local Insurance Committee refused to allow the insured persons the free choice of doctor so often promised them and specifically provided for them by Clause 15 of the Insurance Act. But under the autocratic rule of the Insurance Commissioners the local body, toeing the line with other Insurance Committees all over the country, refused the choice of any but panel doctors; and the inadequate supply of the latter was supplemented by the importation of outside practitioners into the district. It would have been surprising if in these circumstances the professional and social reception of a newcomer by the established practitioners of the place had been particularly hearty, and it is not to be wondered at if the imported panel doctor at Wisbech experienced difficulty in obtaining such help from the non-panel practitioners as the administration of anæsthetics to his patients. For any such services, however, the help of the other doctors on the panel was available; and, assuming the competence of these gentlemen, it is a little difficult to understand the very doubtful compliment paid to their ability in a press report of October 30, which says, " . . . when he had an operation to perform he could get no one to administer the anæsthetic. He had to fall back at last on a local colleague who was on the panel."

### Wisbech Doctors and Trade Unionism.

The mob which professed sympathy with the deceased doctor showed its feelings by destroying the property and threatening the lives of other practitioners, whose chief offence seems to have been that they were officials of the local branch of the British Medical Association, and had declined service under the Act in accordance with the official decision—which is still the declared policy of the Association—that the terms of service were "derogatory to the profession and against the public interest." It may be assumed that the leaders in the disturbance were staunch trade unionists, but they did not stop to ask what would have happened to the sick persons of Wisbech if the doctors of the town had shown their resentment of imported labour by employing trade-union methods and adopting a "down-tools" policy. The insured persons of the town may well be reminded that fortunately for them their own methods of dealing with disputes have not yet found favour with their medical attendants, and further that any difficulty the insured persons may have experienced in securing medical attendance is due solely to the obstructive tactics of the Commissioners and Insurance Committees. It is announced that a fresh outside practitioner is to be introduced. It would have been more satisfactory to hear that the Insurance Committee intended to act fairly to the public and the profession by giving the free choice of doctor so often promised.

### The Chesterfield Example.

Turning to another matter, it has been so generally agreed that medical practice has deteriorated in quality under the Insurance Act that interest is aroused on reading the statement of the *Lancet's* Special Commissioner that in Chesterfield some of the practioners think panel practice will improve the scientific status of the profession. The explanation which follows, however, gives no suggestion of the vaunted "modern and improved methods of diagnosis," but simply states, in effect, that the previous conditions of contract practice in Chesterfield were so extremely unsatisfactory that the mere fact of an increased remuneration is presumably improving the character of medical practice in the district. The account states: "Thus, formerly—and notably at Chesterfield—it often happened that no record of any sort was kept. When a patient attended he might possibly be recognised as having been before, but there was nothing to recall what he suffered from or what treatment he had received. Now a record has to be kept, and the panel medical man, unlike his club predecessor, really does know something about his patients. Further, he must form a conclusion and state in writing what he thinks is the matter. Formerly, a patient was not even examined till he was so ill as to be able to claim sick allowance from his club. When he was well enough to attend at the surgery and to go to his work a bottle of medicine was given him, and that was all. This was guesswork and not scientific practice. Now, with the more precise records that have to be kept under the Act, greater care is displayed." This is rather "damning with faint praise," and it may be freely admitted that in some localities the level of panel practice would be indeed low did it not effect some improvement on the old conditions.

### Two Officers to 7,000 Members.

In Chesterfield, it appears that the panel doctors are experiencing competition from the Chesterfield District Medical Association, which is an amalgamation of friendly societies and clubs. This amalgamation, having been in existence before the passing of the Insurance Act, is enabled under Section 15, Clause 4, of the Act to employ its own whole-time medical officers. There are two of these officers to give attendance to 7,000 members, and an attempt to secure a third has not yet succeeded. We may safely assume, therefore, that the salaries paid are well below the Insurance Act equivalent; in other words, the case is one of sweating of these medical men. Considerations of space prevent further reference at the moment to the existence of these institutes, which are a menace to the public health and a source of unfair competition between medical men. Being commercial and not purely medical concerns, they are not troubled with ethical considerations, and touting for members is freely carried on. It will be necessary to revert later to a consideration of institutes of this character, for there is a real danger of their extension; and, as is well said by the *Lancet's* Commissioner, the Insurance Act, "instead of putting an end to the abuses that existed under the old club system, allows . . . the same conditions to grow up again and with increased intensity."

